# Role of macrophages and activated microglia in neuropathic pain associated with chronic progressive spinal cord compression

**DOI:** 10.1038/s41598-019-52234-1

**Published:** 2019-10-30

**Authors:** Naoto Takeura, Hideaki Nakajima, Shuji Watanabe, Kazuya Honjoh, Ai Takahashi, Akihiko Matsumine

**Affiliations:** Department of Orthopaedics and Rehabilitation Medicine, Faculty of Medical Sciences, University of Fukui, 23-3 Matsuoka Shimoaizuki, Eiheiji, Fukui 910-1193 Japan

**Keywords:** Neurological disorders, Neurological disorders, Immunopathogenesis, Inflammation, Neuroscience, Diseases of the nervous system, Neuroimmunology, Somatosensory system

## Abstract

Neuropathic pain (NeP) is commonly encountered in patients with diseases associated with spinal cord damage (e.g., spinal cord injury (SCI) and compressive myelopathy). Recent studies described persistent glial activation and neuronal hyperactivity in SCI, but the pathomechanisms of NeP in chronic compression of the spinal cord remains elusive. The purpose of the present study was to determine the roles of microglia and infiltrating macrophages in NeP. The study was conducted in chimeric spinal hyperostotic mice (*ttw*/*ttw*), characterized by chronic progressive compression of the spinal cord as a suitable model of human compressive myelopathy. The severity of spinal cord compression correlated with proportion of activated microglia and hematogenous macrophages. Spinal cord compression was associated with overexpression of mitogen-activated protein kinases (MAPKs) in infiltrating macrophages and reversible blood-spinal cord barrier (BSCB) disruption in the dorsal horns. Our results suggested that chronic neuropathic pain in long-term spinal cord compression correlates with infiltrating macrophages, activated microglial cells and the associated damage of BSCB, together with overexpression of p-38 MAPK and p-ERK1/2 in these cells. Our findings are potentially useful for the design of new therapies to alleviate chronic neuropathic pain associated with compressive myelopathy.

## Introduction

Neuropathic pain (NeP) represents pain associated with anatomical or functional abnormalities of the nervous system^1^, and is often observed in certain diseases including iatrogenic conditions affecting the somatosensory pathways in the peripheral or central nervous system^2^. For example, about half of patients with spinal disorders suffer from NeP^3^, compared to about 10–30% of those with herpes zoster, ~10–25% of diabetic neuropathy and ~10% with stroke^4–6^. In our recent nationwide survey of patients with spinal cord-related pain syndrome, we introduced the term *spinal cord-related pain syndrome* to define chronic NeP associated with spinal cord damage, and reported its prevalence to be highest in patients with cervical spondylotic myelopathy, followed by spinal cord injury (SCI) and cervical spine ossification of longitudinal ligament (OPLL)^7^. In another multi-center study, we found significantly lower SF-36 scores in patients with spinal cord-related pain syndrome relative to the national average^8^. Our findings suggest that chronic NeP affects the physical and functional status, as well as the mental health and activities of daily living^9,10^, and that the management of chronic NeP requires a multidisciplinary approach.

While the underlying mechanisms of chronic NeP are multifactorial and change with time, spinal and supraspinal lesions are the main mechanisms of NeP. Whereas several studies analyzed the pathomechanism of NeP after SCI, little is known about these mechanisms in patients with compressive myelopathy. Evidence suggests that monocytes, macrophages, and especially glial cells may play important roles in chronic NeP associated with compressive myelopathy^11^. The microglia-specific molecules, P2X purinoceptor 4 (P2X4) and p38 mitogen-activated protein kinases (p38 MAPKs), are upregulated and activated in NeP after peripheral nerve injury^12–14^. In this context, we reported previously that transplantation of bone marrow-derived mesenchymal stem cells (BMSC) reduced NeP after SCI by suppressing the expression levels of PKC-γ, p-CREB, p-p38 MAPK, and p-ERK1/2 in dorsal horn neurons and restoring abnormal blood-spinal cord barrier (BSCB), mediated through modulation of spinal-resident microglia and hematogenous macrophages activity and recruitment^15^. However, there is no information on the effects of progressive compression of the spinal cord on NeP-related pathological changes, such as glial activation and BSCB dysfunction. In this regard, our group published a series of studies conducted in mice with spontaneous spinal cord compression (tip-toe walking mouse (*ttw*/*ttw*)). Some of the main findings of these studies included a profound decrease in the number of neurons in the anterior horn (which correlated with the extent of spinal cord compromise) and increased number of glial cells in both the gray and white matters^16^.

The present study was designed to explore the potential mechanism of NeP in chronic progressive compressed spinal cord using *ttw*/*ttw* mouse. Specifically, we analyzed the status of microglia/macrophage accumulation and MAPK signaling in the compressed areas. Furthermore, we used chimeric *ttw*/*ttw* mice. The bone marrow of this mouse contains green fluorescent protein (GFP)-expressing hematogenous cells. We determined the pathological roles of cervical spinal microglia and macrophages of bone marrow origin in NeP associated with long-term spinal cord compression.

## Results

### MRI assessment of progressive compression of the spinal cord

**Figure 1:**
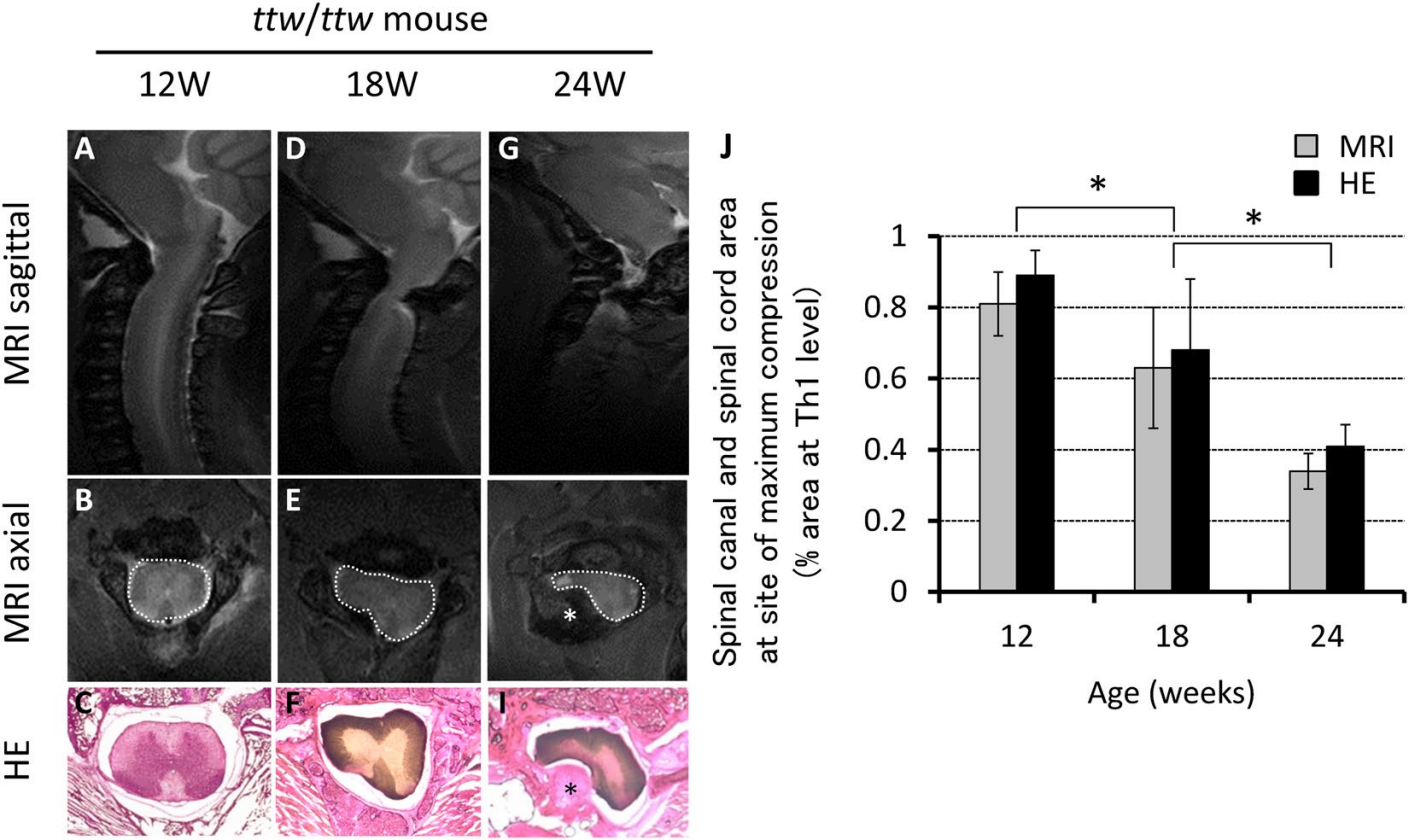
*Left:* Transverse area of the cervical spinal canal. *Right:* Quantification of the transverse area of the spinal canal relative to that at the thoracic (Th) 1 vertebra assessed by MRI (spinal canal transverse area is surrounded by white dotted line). Data are mean ± SD. **p < 0.01, by ANOVA followed by Tukey’s post hoc analysis (n = 3 for each time point). (**B,E,H**). *Top left:* MRI of the cervical spine of 12- (**A,B**), 18- (**D,E**) and 24-week-old (**G,H**) *ttw*/*ttw* mice. *Bottom left*: Microphotographs of hematoxylin and eosin (H&E) stained transaxial sections (**C,F,I**). Note the age-related increase in the size of the calcified lesions originating from the atlantoaxial membrane, resulting in the compression of the lateral and dorsal aspects of the spinal cord between C2 and C3 segments (*) calcified lesions. The spinal canal transverse area also decreased with advancing age (**J**). ttw; tip-toe walking mouse.

Serial analysis showed age-related increase in the severity of spinal cord compression in the *ttw/ttw* mice at the C1-C2 vertebral level; the calcified mass increased in size with age particularly in the atlantoaxial membrane posteriorly. Quantitative analysis of the MRI images and H&E stained sections demonstrated a significant age-related decrease in the C1-C2 spinal cord transverse area, relative to that at the Th1 vertebral level: 0.81 ± 0.09 in 12-week-old, 0.63 ± 0.17 in 18-week-old, 0.34 ± 0.05 in 24-week-old *ttw/ttw* mice (Fig. 1). The above results demonstrated a close correlation between MRI and histological findings.

### Mechanical allodynia and thermal sensitivity in *ttw*/*ttw* mice

**Figure 2:**
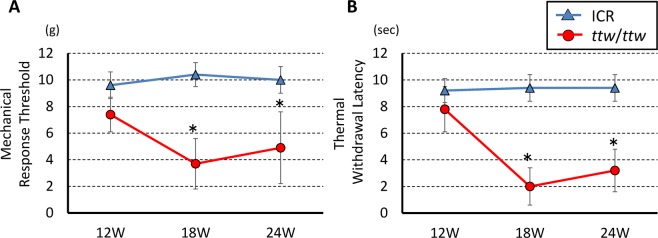
Chronic compression of the spinal cord was associated with hypersensitivity to mechanical and thermal stimulation in ICR and *ttw*/*ttw* mouse at 12-, 18- and 24-week-old (ICR, n = 9; *ttw*/*ttw*, n = 15; *p < 0.05). (**A,B**) 18-week-old ICR mouse as representative. Data are mean ± SD.

The threshold of mechanical and thermal sensitivity scores were significantly lower in *ttw*/*ttw* mice compared with ICR mice at 18- and 24-weeks of age (Fig. 2). In the present study, allodynia was tested in 139 *ttw*/*ttw* mice and 108 (77.7%) of these mice were chosen for the test based on the presence of significant sensory differences at 18- and 24-weeks of age relative to the ICR mouse.

### Expression of GFP in spinal cord of bone marrow-chimeric *ttw*/*ttw* mice

**Figure 3:**
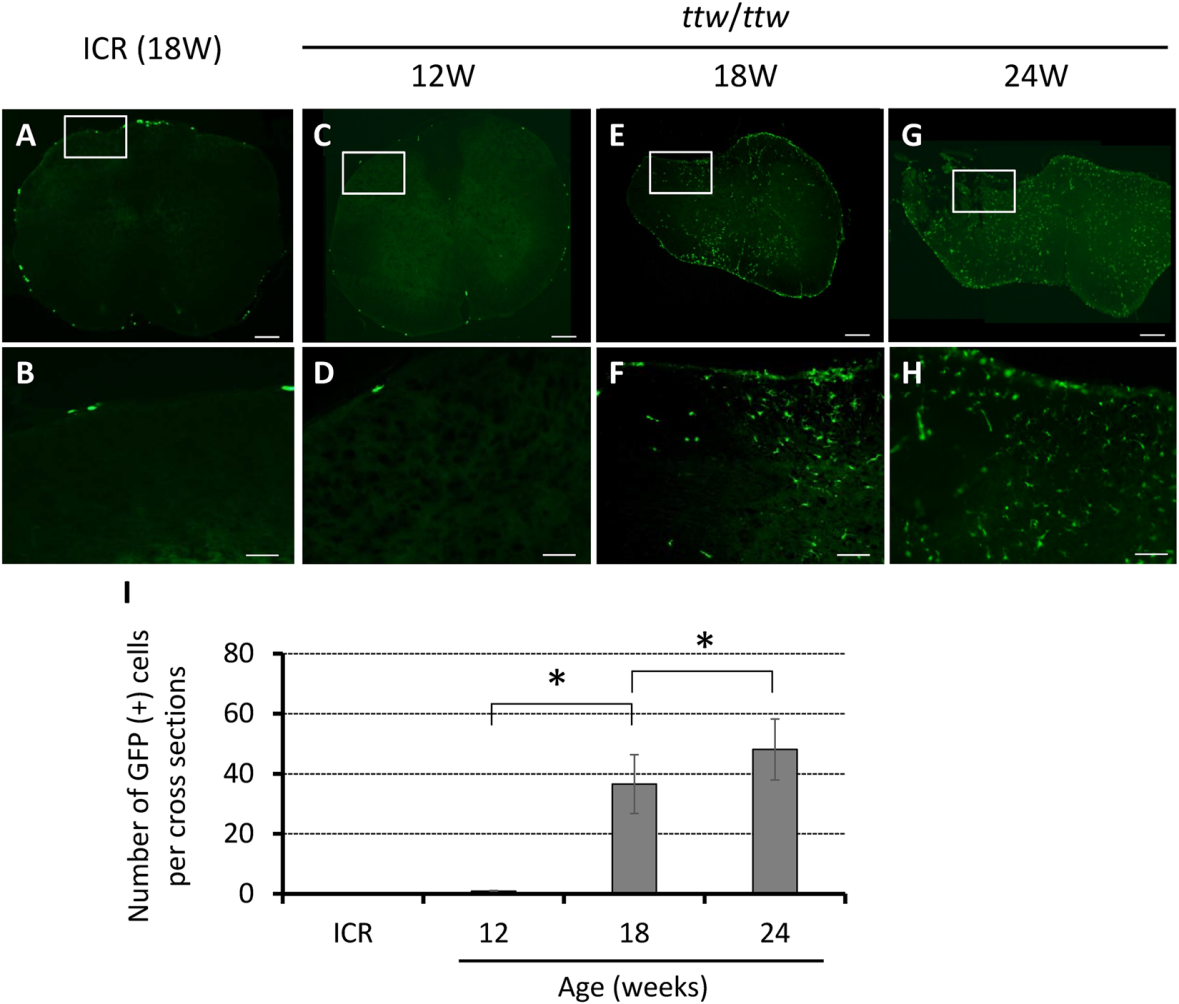
Proportionate increase in GFP-positive cells in spinal cord with age and severity of spinal cord compression. There were virtually no GFP-positive cells in spinal cord of ICR (**A,B**; 18-week-old as representative) and 12-week-old *ttw*/*ttw* mouse (**C,D**), and their appearance in 18-, 24-week-old *ttw*/*ttw* mouse (**E–H**). Note the increase in the number of GFP-positive cells with age of *ttw*/*ttw* mouse (**I**). Scale bars, 200 µm (**A,C,E,G**); 100 µm (**B,D,F,H**). Data are mean ± SD. **p < 0.05 by ANOVA followed by Tukey’s post hoc analysis (n = 5 for each time point). ttw; tip-toe walking, GFP; green fluorescent protein.

To determine whether the bone marrow-derived cells are recruited into the chronically compressed spinal cord, chimeric *ttw*/*ttw* mice were prepared with GFP-labeled hematogenous cells. GFP-positive cells increased significantly in proportion with the degree of spinal cord compression, especially in 18- and 24-week-old mice. There were few GFP-positive cells in ICR and 12-week-old mice (Fig. 3).

**Figure 4:**
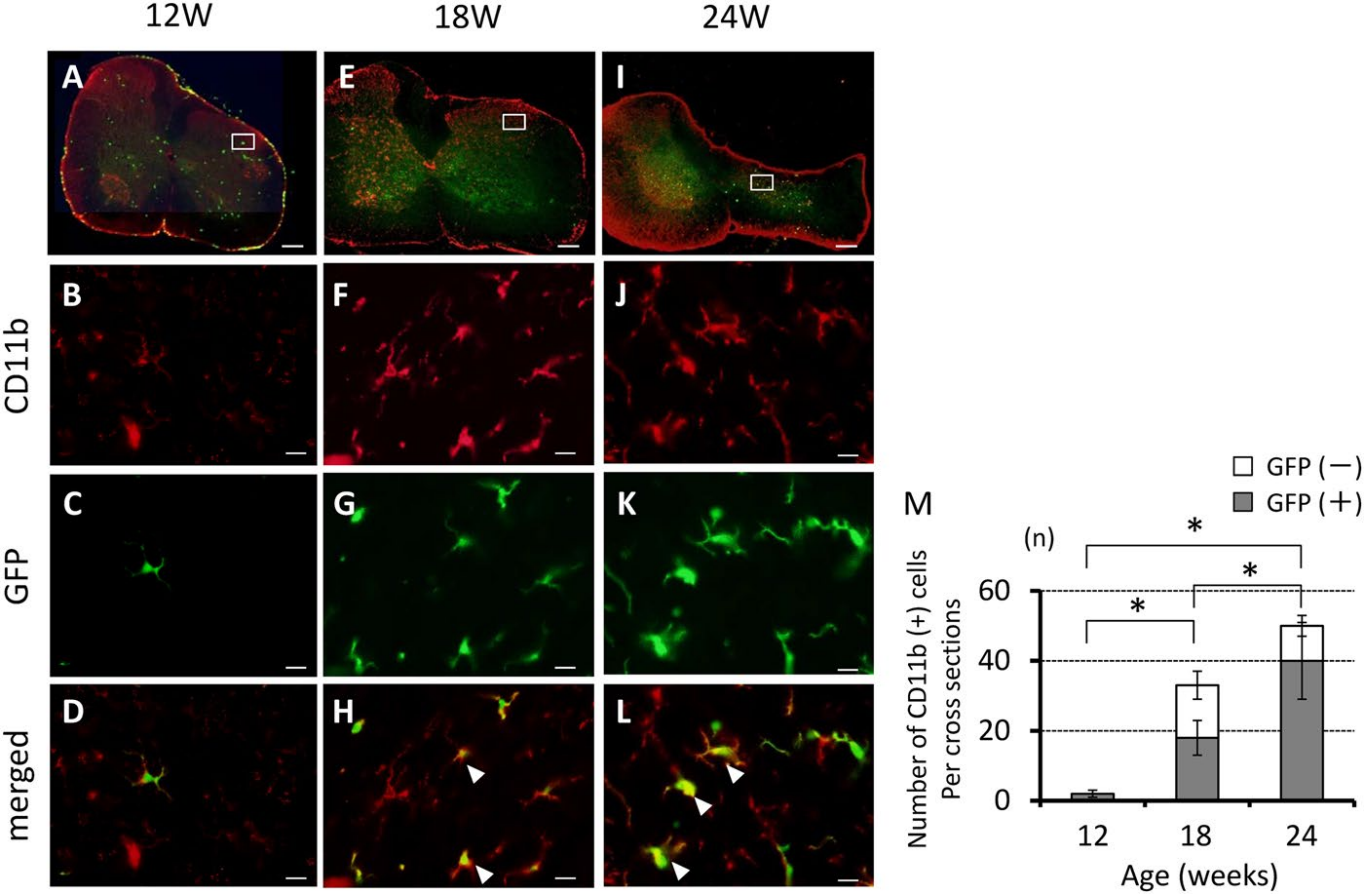
Correlation between neuronal changes in *ttw*/*ttw* mouse and serial changes in the density of activated microglia/macrophages in spinal cord compression. Immunofluorescence staining for the expression of CD11b (red) and GFP in 12- (**A**–**D**), 18- (**E**–**H**) and 24-week-old (**I**–**L**) *ttw*/*ttw* mouse. The number of CD11b-positive cells increased with age and worsened with the severity of spinal cord compression, both in the gray and white matters (**A,E,I**). The number of GFP cells in the gray matter was especially higher in 18- and 24-week-old *ttw*/*ttw* mice (**E,I**). The number of GFP-positive/CD11b-positive cells per cross-section of the posterior horn of the spinal cord was significantly higher in 18- and 24- than 12-week-old *ttw*/*ttw* mice (**M**). Scale bars, 200 µm (**A,E,I**); 50 µm (**B–D,F–H**,**J–L**). Data are mean ± SD. **p < 0.01, by ANOVA followed by Tukey’s post hoc analysis (n = 5 for each time point).

Immunostaining of transverse sections of the spinal cord of *ttw*/*ttw* mice showed a proportionate increase in the number of CD11b positive cells in the dorsal horn with the degree of spinal cord compression. Dual-labeled cells for CD11b and GFP also increased proportionately with the degree of spinal cord compression and the proportion of CD11b and GFP dual labeled cells was high among CD11b-positive cells (Fig. 4). Figure 4M shows the number of activated microglia (CD11b-positive/GFP-negative cells) and hematogenous macrophages (CD11b-positive/GFP-positive cells) in the dorsal horn. The percentage of hematogenous macrophages among CD11b-positive cells increased significantly from 51.3 ± 3.9% in 18-week-old to 80.3 ± 8.7% in 24-week-old *ttw/ttw* mice.

**Figure 5:**
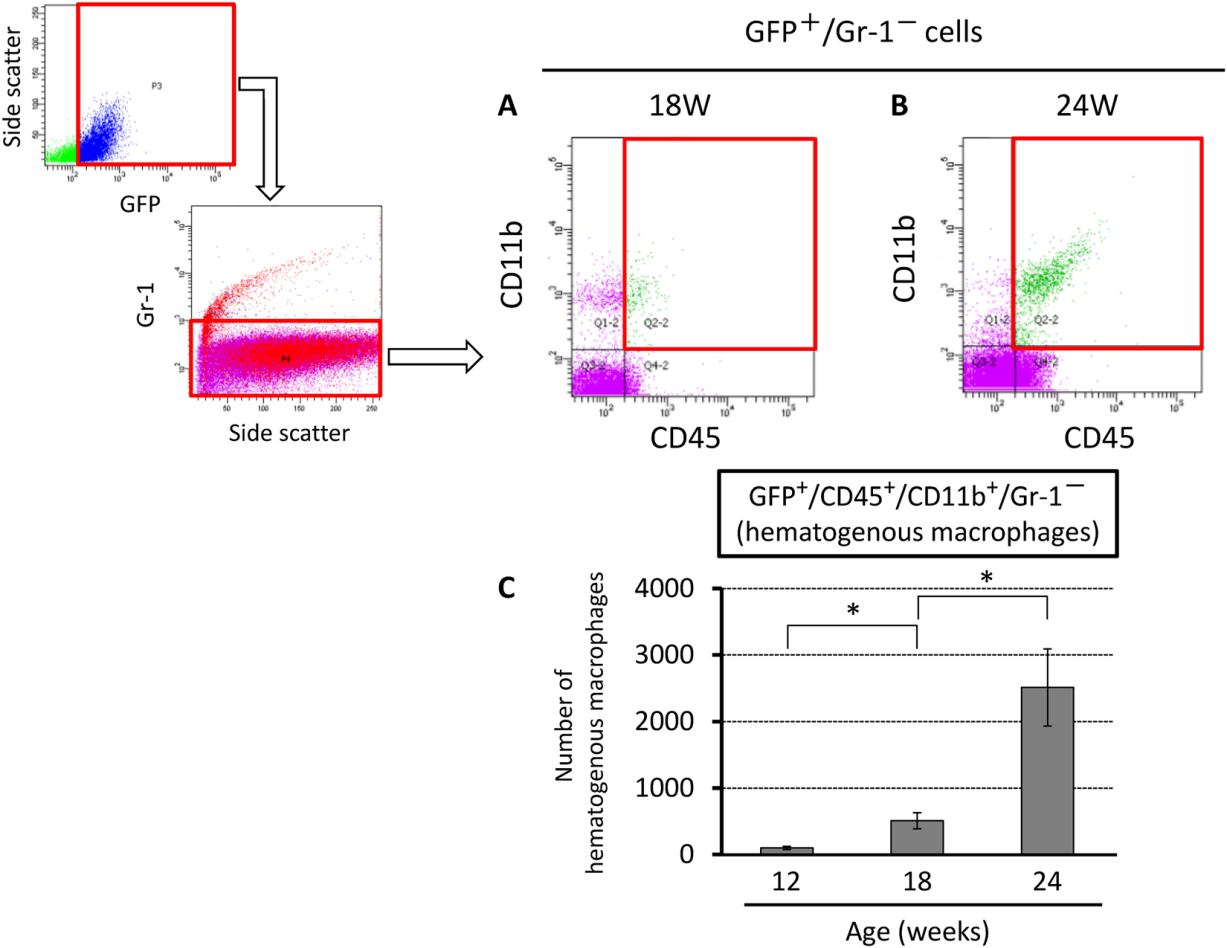
Proportionate increase in the percentages of hematogenous macrophages with age and severity of spinal cord compression in *ttw*/*ttw* mouse. Semi-quantitative flow cytometric analysis of hematogenous macrophages and activated microglia/macrophages according to the degree of spinal cord compression in representative 18- (**A**) and 24-week-old (**B**) *ttw*/*ttw* mice. The number of hematogenous macrophages (CD11b positive/GFP positive/CD45 high/GR-1negative cells) was significantly higher in 18- and 24-week-old mice (**C**). Data are mean ± SD. **p < 0.01, by ANOVA followed by Tukey’s post hoc analysis (n = 3 for each time point).

To examine the recruitment of hematogenous macrophage into the chronic compressed spinal cord, GFP^+^/CD45^+^/CD11b^+^/Gr-1^−^ cells were quantified by flow cytometric analysis. The number of GFP^+^/CD45^+^/CD11b^+^/Gr-1^−^ cells per cross section increased significantly and gradually in the chronically compressed spinal cord of *ttw*/*ttw* mice from 100 ± 24 in 12-week-old, to 509 ± 122 in 18-week-old and 2512 ± 578 in 24-week-old mouse (Fig. 5).

### Expression of MAPK-positive cells in dorsal horn

**Figure 6:**
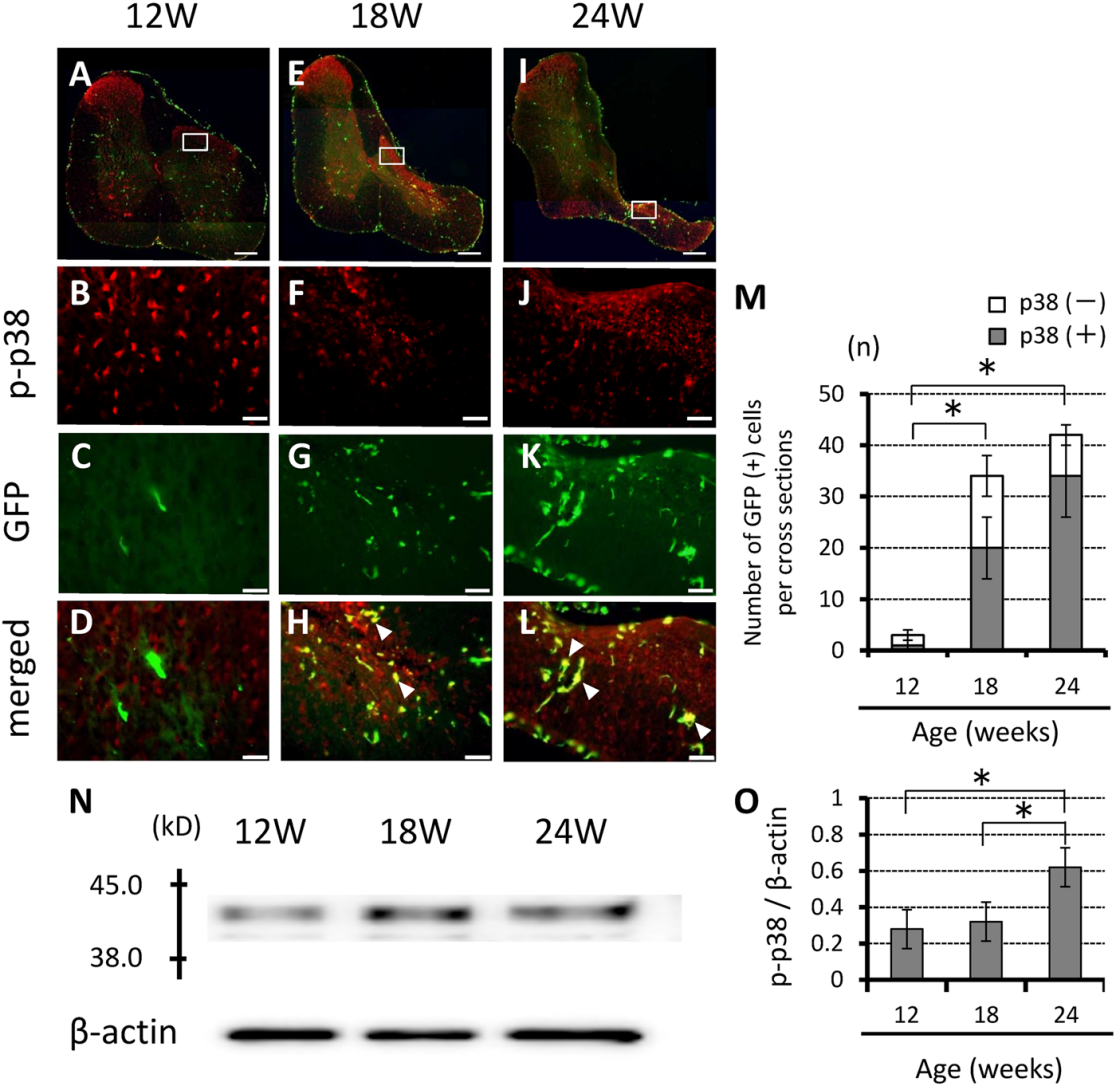
Chronic compression of the spinal cord is associated with increased number of p-p38 MAPK-positive GFP cells. (**A–L**) Representative axial immunofluorescence staining showing colocalization of p-p38 MAPK with GFP in18- and 24-week-old mice. The number of these cells was higher, especially in the spinal dorsal horn (**A,E,I**). (**M**) Number of GFP-positive cells. Scale bars, 200 µm (**A,E,I**); 50 µm (**B–D,F–H,J–L**). Data are mean ± SD of 3 mice per age group. Note the age-related increase in the number of p-p38-positive cells among GFP-positive cells. (**N**) Western blotting showed significantly higher p-p38 MAPK protein levels in 18- and 24-week-old *ttw*/*ttw* mice compared with their 12-week-old counterparts. (**O**) Relative band intensity of p-p38 normalized to that of β-actin (n = 3 each; *p < 0.05). MAPK; mitogen activated protein kinase.

**Figure 7:**
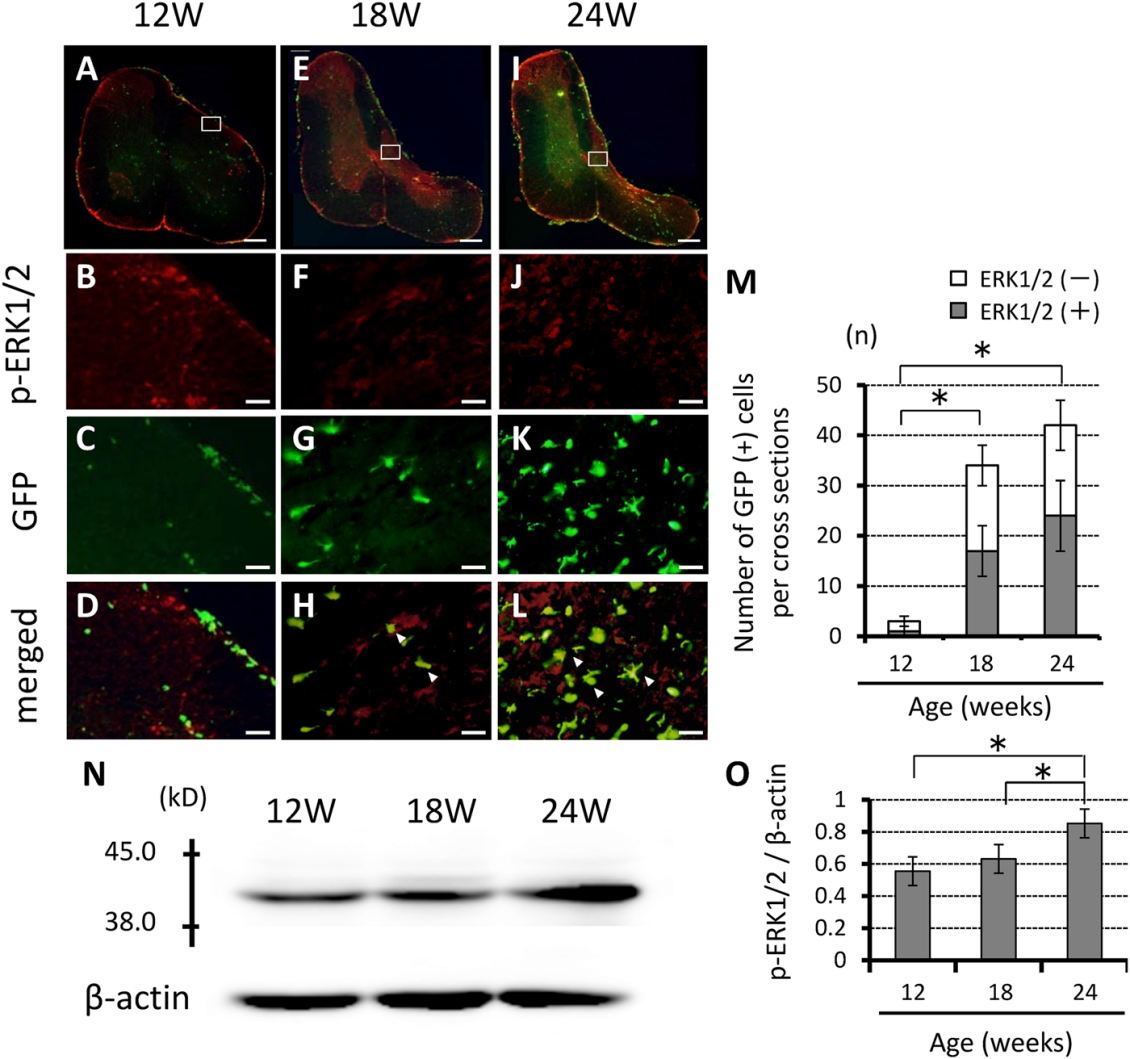
Chronic compression of the spinal cord was associated with increased numbers of p-ERK1/2 MAPK-positive GFP cells. (**A–L**) Representative axial immunofluorescent staining showing colocalization of p-ERK1/2 MAPK with GFP in 18- and 24-week-old mice. The number of p-ERK1/2 MAPK colocalized with GFP immunolabeled cells was higher, especially in the spinal dorsal horn (**A,E,I**). (**M**) Number of GFP-positive cells. Scale bars, 200 µm (**A,E,I**); 50 µm (B-D, F-H, J-L). Data are mean ± SD of 3 mice per age group. Note the age-related increase in the number of p-ERK1/2-positive cells among GFP-positive cells. (**N**) Western blotting showed significantly higher p-Erk1/2 MAPK protein levels in 18- and 24-weeks-old *ttw*/*ttw* mice compared with their 12-week-old counterparts. (**O**) Relative p-Erk1/2 MAPK band intensity normalized to that of β-actin (n = 3 each; *p < 0.05). MAPK; mitogen activated protein kinase. ERK; extracellular signal-regulated kinase.

Chronic spinal cord compression in *ttw*/*ttw* mice was associated with increases in dually labeled p-p38 MAPK/GFP and p-ERK1/2/GFP cells within the spinal cord dorsal horn (Figs 6 and 7). The number of p-p38-positive cells among GFP-positive cells increased significantly with worsening of spinal cord compression, from 0.67 ± 0.82 in 12-week-old, 20.50 ± 11.52 in 18-week-old, to 34.50 ± 10.05 in 24-week-old *ttw*/*ttw* mice (Fig. 6M). A similar trend was noted with p-ERK1/2 cells among GFP-positive cells (from 0.67 ± 0.82 in 12-week-old, 16.67 ± 8.85 in 18-week-old, to 24.67 ± 12.47 in 24-week-old *ttw*/*ttw* mouse) (Fig. 7M). The correlation between severity of compression of the spinal cord and p-p38 MAPK and p-ERK1/2 was evaluated by western blotting. The intensities of the p-p38 band increased with age; which were significantly higher at 24 weeks than at 12- and 18 weeks (Figs 6N,O and 7N,O).

### Effects of chronic spinal cord compression on BSCB function

**Figure 8:**
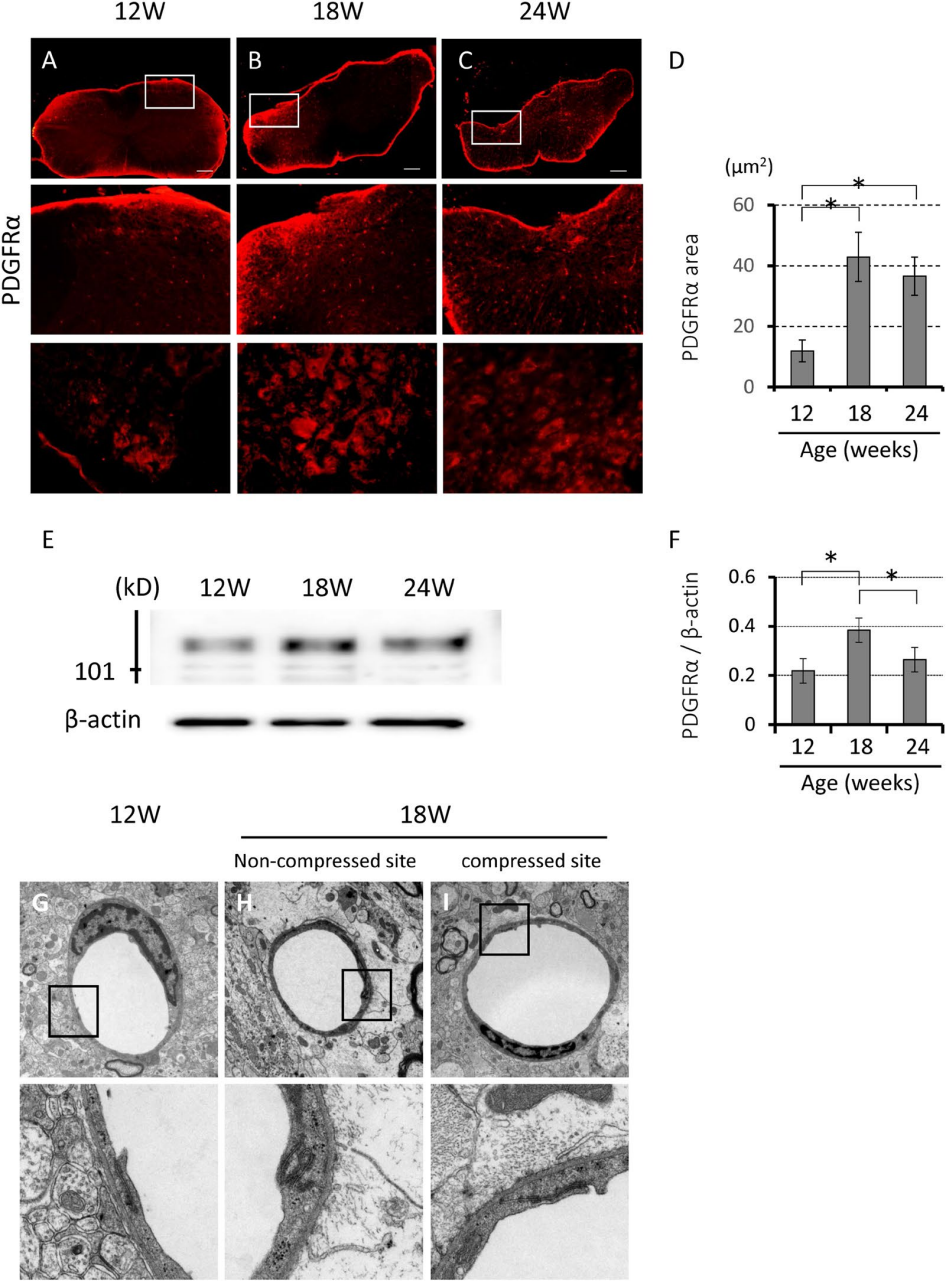
Chronic compression of the spinal cord was associated with disruption of the BSCB. (**A**–**C**) Representative immunofluorescent staining around the site of chronic compression showing the presence of PDGFR-α in the spinal cord of 18- and 24-week-old *ttw/ttw* mice. There was little evidence for PDGFR-α expression in moderate and sever compression of the spinal cord. The boxed areas in the top raw images were highly magnified in the middle and bottom rows. Scale bars 200 *(Top)*, 100 *(Middle)*, 50 *(Bottom)* μm (**A–C**). The PDGFR-α-positive areas in the gray matter were significantly higher in the 18-week-old *ttw*/*ttw* mouse (**B**) compared with their 12-week counterparts (**A**). The PDGFR-α-positive area was slightly smaller in 24-week-old *ttw/ttw* mice (**C**) compared with 18-week-old *ttw*/*ttw* mice (n = 3 each; *p < 0.05). (**D**) Semi-quantitative analysis of the PDGFR-α-positive area in the dorsal horn according to age of the *ttw/ttw* mouse. *p < 0.05, (**E**) Western blotting showed significantly higher PDFGR-α levels in 18-old *ttw/ttw* mice compared with their 12- and 24-week-old counterparts. **(F**) Relative band intensity of PDGFR-α normalized to that of β-actin (n = 3 each; *p < 0.05). one-way factorial analysis of variance. (**F–H**) Ultrastructure of the BSCB examined by transmission electron microscopy in the dorsal horn of 12- (**G**) and 18-week-old *ttw*/*ttw* mouse (**H**: non-compressed site, **I**: compressed site): note the lack of destruction of blood vessel structure in both 12- and 18-week-old *ttw*/*ttw* mice (n = 3each). BSCB; blood-spinal cord barrier. PDGFR-α; platelet-derived growth factor receptor α.

In this study, the expression of PDGFR-α, which is localized on perivascular astrocytic endfeet was used as a surrogate marker of BSCB function. PDGFR-α immunoreactivity at the lesion site increased significantly with chronic spinal cord compression from 12 to 18 weeks of age. However, it was lower in 24-week-old, compared with the that in 18-week-old *ttw*/*ttw* mice (Fig. 8A–F). Transmission electron microscopy indicated that endothelial cells, pericytes and astrocytic endfeet were the main structures that formed blood vessels in the spinal cord. Although the expression of PDGFR-α increased with spinal cord compression, there was no marked disruption of the vascular structures at the dorsal horn in 18-week-old *ttw*/*ttw* mouse (Fig. 8G–I).

## Discussion

Little is known about the mechanisms of NeP in chronic progressive spinal cord compression, compared with that of peripheral nerves, especially with regard to the roles of microglial activation and macrophages infiltration. NeP is not just a symptom of disease, but a consequence of disordered functioning of the nervous system^11^. The pathogenesis of NeP and central sensitization after peripheral nerve injury and traumatic SCI seems to involve spinal cord microglia and macrophages^17,18^. The aim of this research was to determine the roles of activated microglia and infiltrating macrophages in NeP associated with chronic progressive spinal cord compression. In the present study, GFP-labeled bone marrow chimeric *ttw*/*ttw* mice were used to determine the roles of these cells in NeP associated with long-term compression of the spinal cord. The main novel findings of the present study were activated microglia accumulation and macrophage migration from the peripheral circulation into the neural tissue through temporary disruption of the BSCB, together with increased activation of the MAPK signaling pathway. The study showed that these changes varied according to the severity of spinal cord compression, suggesting the involvement of these mechanisms in NeP.

Is the *ttw/ttw* mouse a suitable animal model for investigating the effects of chronic progressive compression of the spinal cord? The histopathological and pathophysiological changes found in these mice are significantly similar to those usually found in traumatic SCI. They include fewer neuronal cells, degeneration and demyelination of neurons, and various secondary changes. Cadaver studies that examined the spinal cords of patients with cervical spondylosis or OPLL reported that chronic progressive mechanical compression of the spinal cord initially induces hypoxia/ischemia and is characterized by the loss and exfoliation of anterior horn neurons with progressive spongy degeneration and demyelination in the white matter^19,20^. These changes in turn induce a variety of extracellular and intracellular pro-apoptotic pathways and neuroinflammation. It seems that these cellular changes contribute to the neurological functional deficits and NeP observed in cervical myelopathy^21^. In a series of studies using the *ttw*/*ttw* mouse, we reported previously that severe spinal cord compression correlated significantly with fewer remaining surviving neurons and severe demyelination of the white matter^16,22^. These findings were similar to those seen in cadaver studies, indicating that the *ttw*/*ttw* mouse is an appropriate animal model for investigating the effects of long-term mechanical compression of the spinal cord.

Our study confirmed the roles of activated microglia and macrophages in the pathogenesis of NeP in chronic compression of the spinal cord. Glial cells are considered to play an important role in the maintenance of neuronal function. However, recent studies demonstrated the involvement of these cells in neuroplasticity. In particular, activated microglia and macrophages in the spinal cord are involved in the pathomechanisms of NeP. Recent studies demonstrated that traumatic injury of the neural system, such as SCI, induces neuronal hyperactivity and persistent glial activation, the two important substrates of central NeP^23^. The activated microglia and recruited macrophages are thought to play important roles in neuroinflammation through the induction/modulation of a wide range of cellular responses^24^. Neuroinflammation plays an important role in the establishment and maintenance of both NeP and chronic pain. Increased levels of inflammatory cytokines, chemokines and microglial activation contribute to the activation of pain mechanisms; chronic pain is the result of dysregulated glial activation^25–27^. The mechanism of NeP that follows SCI includes activation of various MAPK family members; both p-38 MAPK and ERK are activated in microglia in the spinal dorsal horn, which play a role in the development and persistence of NeP, which enhances central sensitization and long-term potentiation^28,29^. These kinases play a key role in microglial signaling since the latter can be activated by multiple microglial receptors, and it also regulates the synthesis of many inflammatory mediators associated with pain facilitation. We reported previous study that transplantation of mesenchymal stem cells soon after SCI relieved pain hypersensitivity through inhibition of MAPK signaling pathway and lessened recruitment of inflammatory cells^15^. Other studies reported that activated microglia play important roles in both hindpaw behavioral sensitivity and lumbar neuronal hyperexcitability via activation of p38 MAPK and ERK1/2 phosphorylation^30,31^. On the other hand, little is known about the pathomechanism of chronic NeP in patients with compressive myelopathy, especially whether hematogenous macrophages play a role in NeP associated with chronic spinal cord compression. In this study, GFP-labeled bone marrow chimeric *ttw*/*ttw* mice were used to determine the relative roles of these cells since activated microglia and macrophages in the injured CNS cannot be distinguished by their morphology or the use of antigenic makers. Our results indicated that the density of activated microglia/macrophage (CD11b^+^ cells) in the dorsal horn was proportionate with the severity of spinal cord compression, hematogenous macrophages (CD11b and GFP double-positive cells) constituted 51.3% in 18- and 80.3% in 24-week-old *ttw/ttw* mice of the cells. In addition, many of these macrophages expressed p-38 MAPK and ERK1/2, which are known to play a role in the expression of mechanical allodynia. It is likely that high-dose irradiation plays a negligible role in the disruption of the BSCB since only a few GFP-positive cells were found in ICR and 12-week-old *ttw*/*ttw* mice.

Another finding of our study was increased BSCB permeability in the chronically compressed spinal cord. Following SCI, damage to endothelial cells from the resultant ischemia may alter BSCB integrity and influx of inflammatory cells and immunocytes to the inflammatory response site around the site of injury. Thus; the spinal cord microglia and macrophages are probably involved, at least in part, in the pathogenesis of NeP and central sensitization after SCI^15^. Increased BSCB permeability alters the cellularity of the spinal cord microenvironment and this could further potentiate neuroinflammation^32^. Little information is available on the mechanisms of BSCB dysfunction and neuroinflammatory responses in animal models with chronically compressed spinal cord. In our study, a significant increase in PDGFR-α immunoreactivity was noted at the lesion site especially in 18-week-old *ttw/ttw* mice. The results suggest that both chronic compression and traumatic injury of the spinal cord enhance BSCB permeability. Interestingly, marked disruption of the vascular structures of the dorsal horn was not observed in the present study, in contrast to traumatic spinal cord injury. Our results also suggested that the increased BSCB permeability was reversible in our model. A previous study using gadolinium-diethylenetriamine pentaacetic acid (Gd-DTPA) enhancement magnetic resonance imaging showed disruption of the spinal cord parenchyma and disturbance of the BSCB in the injured spinal cord, suggesting that the enhancement disappeared or decreased after surgery in most patients with cervical myelopathy; and that disturbance of BSCB in chronic compression can be reversible^33^.

In summary, the results of this study suggested that progressive long-term spinal cord compression sets up a chain of events characterized by macrophage migration from the peripheral circulation into the neural tissue through temporary disruption of the BSCB as well as increased microglia activation, which was proportionate with the severity of compression. These results suggest that NeP is mediated through these histopathological changes. Our findings are potentially useful for the design of new therapies that can alleviate NeP by reducing neuroinflammation through the suppression of microglial activation and/or macrophage migration.

## Methods

All methods in this study were performed in accordance with the relevant institutional guidelines and regulations.

### Animals

The study was conducted in spinal hyperostotic *ttw/ttw* mice (12-week-old, n = 37, 18-week-old, n = 37, 24-week-old, n = 34), with a mean body weight of 28.3 ± 1.5 g, purchased from the Central Institute for Experimental Animals (Kawasaki, Japan). Homozygous *ttw/ttw* mice were naturally occurring mutant mice and maintained by brother-sister mating of heterozygous ICR mice (+/*ttw*, Clea, Tokyo, Japan). ICR mice of the same age were used as the control group (n = 9). Hyperostosis is induced under an autosomal recessive condition and the homozygous hyperostotic mouse assumes a tip-toe walking pattern at the age of 6–8 weeks, although no congenital neurological abnormalities are detected at that age. In the *ttw/ttw* mouse, calcified masses appear at the posterior aspects of C1 and C2, causing C2 and C3 cord segment compression with ankylosis. The calcified masses increase in size with age particularly in the atlantoaxial membrane, and often produce extensive motor weakness in 18–24-week-old mice^34,35^. Adult male mice (age 6–8 weeks) weighing 19.2 ± 0.6 g show upregulation of enhanced-GFP (EGFP), which is induced by CAG (cytomegalovirus early enhancer β-actin) transgene (CAG-EGFP mice; Nihon SLC, Shizuoka, Japan).

Following institutional ethical review and approval (The Institutional Animal Care and Use Committees of Fukui University, Department of Orthopaedics and Rehabilitation Medicine: Approval Number 23–002) and the Ethical Guidelines of the International Association for the Study of Pain, all mice were handled carefully to lessen any chance of pain or discomfort.

### Hematoxylin and eosin staining

The cervical spine (n = 5 for each time point) was resected carefully as described previously and then fixed in buffered formaldehyde for 48 hours at 4 °C^34^. Later, the obtained tissue was decalcified in 0.5 M ethylenediaminetetraacetic acid (0.5 M Tris-HCl buffer, pH 7.6) over a period of 2 weeks at 4 °C. The sections were embedded in paraffin and later cut into 20-µm-thick sagittal and axial sections using a cryostat, which were then stained with hematoxylin and eosin (H&E).

### Assessment of progressive compression of spinal cord with MRI

The severity of compression of the cervical spine was assessed by measuring the spinal canal area using 7T-magnetic resonance imaging (MRI) (BioSpec^®^, Bruker biospin, Billerica, MA) in anesthetized animals. The obtained images were assayed using Image J analysis software (NIH, Bethesda, MD). The analyzed the correlation between age and spinal canal area measured at the most severe level of compression in the C1-C2 region. Furthermore, modifying the methods as described previously^16^, the spinal canal areas at C1 and C2 were compared with that of thoracic (Th) 1 vertebra.

### Sensory testing

Two independent examiners (S.W. and K.H.) who were blinded to the experimental condition in order to avoid any bias tested the mice at different ages (12, 18, and 24 weeks) for mechanical allodynia and thermal sensitivity. The former was tested by the Dynamic Plantar Aesthesiometer (Ugo Basile, Comerio, Italy)^36^. In this test, the withdrawal threshold (expressed in grams) is determined five times and the mean value is reported. The independent examiners also tested for thermal sensitivity at the plantar hindpaws using the Plantar Test Apparatus (Ugo Basile), as described in detail previously^37^. In this test, the time between application of the thermal stimulus to hindpaw withdrawal (latency) is recorded (in sec), as well as any other reaction to the stimulus (e.g., gazing at the affected paw, sniffing, licking, or attacking the stimulus). The latency was calculated using data of six tests after rejecting the longest and shortest latencies, as described previously by Hoschouer *et al*.^38^.

### Bone marrow-chimeric *ttw*/*ttw* mice

The bone marrow-chimeric *ttw/ttw* mice was generated using highly purified, genetically marked bone marrow cells, as described in detail elsewhere^15,17,39^. Isolated unfractionated marrow cells (5.0 × 10^6^ cells) were obtained from the donor mice (CAG-EGFP transgenic mice) and injected into the tail vein of previously irradiated 9-, 14- and 20-week-old *ttw/ttw* and ICR mice (dose: 9.0 Gy over 30 min, 4.5 Gy/15 min twice). Engraftment and induction of chimerism was confirmed by identifying the donor cells 3–4 weeks later by FACS Calibur (Dual-laser Fluorescence-Activated Cell Sorting, BD Biosciences, San Jose, CA). Subsequent experiments were conducted in 12-, 18- and 24-week-old bone marrow chimeric mice.

### Immunohistochemistry

For immunohistochemical analysis, the mouse was deeply anesthetized (n = 5/time point), transcardially perfused and the obtained tissues were fixed with 4% paraformaldehyde in 0.1 M phosphate-buffered saline (PBS). Similarly, the spinal cord was dissected out carefully and kept in a similar fixative. After 3 hours in the fixative solution, the tissue samples were immersed in a mixture of 10% sucrose/0.1 M PBS and maintained at 4 °C for 24 hours, and then in another solution of 20% sucrose/0.1 M PBS for another 24 hours. On the other hand, the cervical area of the spinal cord was embedded in OCT (optimal cutting temperature) compound (Sakura Finetek, Torrance, CA) and then cut into serial 20 µm-thick axial or sagittal frozen sections using a cryostat. The cut sections were serially mounted on glass slides and fixed for 5 min with 2% paraformaldehyde in 0.1 M PBS, followed by rinsing in PBS and storage under cold temperature (−80 °C).

The immunohistochemical staining continued with permeabilization of the frozen sections with 0.1 M Tris-HCl buffer (with 0.3% Triton X-100, pH of 7.6). The sections were treated overnight with following primary antibodies (Abs) at 4 °C, which were diluted with the Antibody Diluent with Background Reducing Components (Dako Cytomation, Carpinteria, CA): rabbit anti-phospho-p38 MAP kinase (p-p38) polyclonal Ab (dilution, 1:200, Cell Signaling Technology, Beverly, MA); rabbit anti- p44/42 MAPK (p-ERK1/2) polyclonal Ab, 1:200 (Cell Signaling Technology); rabbit anti-integrin αM (or CD11b), 1:200 (Santa Cruz Biotechnology, Santa Cruz, CA). The sections were then incubated with Alexa Fluor-conjugated 488- or 568- secondary antibodies (dilution, 1:250, Molecular Probes, Eugene, OR) for 1 hr at room temperature. Finally, the sections were washed, wet-mounted, and examined by omitting the primary antibody or through the use of a non-specific negative isotype-matched primary antibody, as described previously^16^. The sections were examined under a fluorescence microscope (Olympus AX80, 200 Olympus Optical, Tokyo) or confocal laser scanning microscope (model TCS SP2, Leica Instruments, Nussloch, Germany), using 488- and 543-nm lines of the argon/helium-neon laser for fluorescence excitation.

### Semi-quantitative analysis

At 12, 18, and 24 weeks of age, five axial sections were randomly selected from the site of maximum compression (between C2 and C3 dorsal roots) and half of the spinal cord on the compressed side, to determine the density of GFP^+^ cells, CD11b^+^/GFP^+^ cells, and p-38 MAPK^+^ and p-ERK1/2^+^ colocalized with GFP^+^ cells in the superficial laminae I-III of the spinal dorsal horn. For PDGFR-α staining, we calculated the area and pixel density within the immunoreactivity threshold value in the superficial laminae I-III of spinal dorsal horn on the compressed site, as described previously^2^. The calculated immunopositivity represented the pixel density multiplied by image area. The latter was determined using the color image analyzer (MacSCOPE; Mitani, Fukui, Japan).

### Flow cytometric analysis

Flow cytometric analysis was conducted as described previously^15^ using tissues harvested from 2.5 mm on either side of the site of spinal cord compression in 12-, 18- and 24-week-old bone marrow chimeric mice. Before immunostaining for flow cytometry, cell-count was performed in each sample in order to ensure cell concentration at 1.0 × 10^6^ cells/100 µl. Hematogenous macrophages represented GFP^+^ CD45^high^ CD11b^high^ GR-1^−^ cells while endogenous activated microglia represented GFP^−^ CD45^high^ CD11b^high^ GR-1^−^ cells, as described previously^40^. After sorting GFP-positive cells, intracellular staining was performed as described in detail previously^41^. Briefly, the harvested cells were resuspended in fixation buffer and then treated with permeabilization buffer (Santa Cruz Biotechnology, Santa Cruz, CA). They were then re-suspended in ice-cold PBS and incubated with one of the following Abs: 0.25 µg/1 ml PerCP-Cy^TM^ 5.5 rat anti-CD11b Ab (BD Pharmingen, San Jose, CA), 0.25 µg/1 ml allophycocyanin (APC) rat anti-CD45 Ab (BioLegend, San Diego, CA), and 1.0 µg/1 ml Pacific Blue^TM^ rat anti-Ly-6G/Ly-6C Ab (Gr-1, BioLegend), each for 1 hr. Flow cytometry was then performed using FACS CantoTM II (BD Biosciences) with forward scattering to eliminate any cellular debris.

### Immunoblot analysis

For immunoblot analysis, the spinal cord was carefully dissected *en bloc* from the area of greatest compression (located between the exit of C2-C3 dorsal roots) (n = 3 mice/each time point) and stored at −80 °C, as described previously^16^. The sections were centrifuged at 15,000 × *g* for 30 sec (BioMasher Rapid Homogenization Kit, Funakoshi, Tokyo), then solubilized in RIPA lysis buffer 1 × (Santa Cruz Biotechnology), homogenized and stored at −80 °C. To determine the protein concentration in the tissue samples, we used the DC protein assay kit (Bio-Rad Laboratories, Hercules, CA) according to Lowry protein Assay. The protein mixtures were mixed with Laemmli sodium dodecylsulfate buffer and boiled prior to immunoblot analysis. The total protein (20 µg/lane) was separated on 12.5% SDS-PAGE and transferred onto polyvinylidene difluoride membrane (PE Applied Biosystems, Foster, CA) for 70 min. The membranes were washed twice in PBS solution containing 0.05% Tween 20, then blocked by a mixture of 5% skimmed milk in PBS for 1 hr at room temperature, and finally incubated overnight with one of the following antibodies at 4 °C: rabbit anti-phospho-p38 MAP kinase (p-p38) polyclonal Ab or rabbit anti- p44/42 MAPK (ERK/2) polyclonal Ab (each diluted at 1:200, and both from Cell Signaling Technology, Danvers, MA); or rabbit anti-platelet-derived growth factor receptor-α (PDGFR-α) polyclonal Ab (dilution, 1:200, Santa Cruz Biotechnology). After washing three times in 0.1 M PBS, the membranes were incubated for 1 hr in the respective secondary IgG/HRP complex Abs: anti-goat (dilution, 1:1,000); anti-rabbit (dilution, 1:5,000); or anti-rat (dilution, 1:1,000) (all from Santa Cruz Biotechnology). After washing three times in 0.1 M PBS, the membranes were immersed in ECL Advance Western Blot Detection kit (GE Healthcare, Buckinghamshire, UK) for 1 min and examined with imaging analyzer (Image Quant LAS 4000 mini chemiluminescence, GE Healthcare Life Science, Piscataway, NJ). The intensity of each band was quantified using Image Quant TL software (GE Healthcare Life Science) and expressed relative to that of β-actin. For molecular weight controls, we used the Kaleidoscope Prestained Standards (Bio-Rad Laboratories, Hercules, CA).

### Electron microscopy

Tissues harvested from 12- and 18-week-old *ttw*/*ttw* mice were used for electron microscopic examination. Briefly, the mouse was euthanized by deep anesthesia, then fixed with 2.5% glutaraldehyde and 2.5% paraformaldehyde, then fixed with 1% osmium tetroxide for 2 hrs. The spinal cord area around the site of maximum compression was removed and the fixed specimens were dehydrated in a graded series of alcohol, embedded in epoxy resin and polymerized at 60 °C for 2 days. Ultrathin sections were obtained using an ultramicrotome and stained with uranyl acetate and lead citrate. Finally, images were prepared using a transmission electron microscope (model H-7650 TEM; Hitachi, Tokyo).

### Statistical analysis

All values are expressed as mean ± SD. Differences between groups were tested for statistical significance using one-way analysis of variance (ANOVA). The *p* value determined by Tukey’s post hoc analysis was set to <0.05. For measurement of stained tissues, the inter- and intra-observer reliability was assessed by calculating intraclass correlation coefficients (ICC), and ICC values < ± 0.75–1.00 were considered to represent excellent reliability. All statistical tests were performed using SPSS software version 24.0 (SPSS, Chicago, IL).

## Data Availability

Data generated and analyzed during this study are included in this published article. Data and materials are available from the corresponding author subject to reasonable request and subject to the ethical approvals in place and materials transfer agreements.
